# Mitigating COVID-19 in meat processing plants: what have we learned from cluster investigations?

**DOI:** 10.3389/fpubh.2024.1432332

**Published:** 2024-09-02

**Authors:** Pauline Kooh, Yvonnick Guillois, Michel Federighi, Mathilde Pivette, Anne-Laure Maillard, Ngoc-Du Martin Luong, Estelle Chaix

**Affiliations:** ^1^Risk Assessment Department, French Agency for Food, Environmental and Occupational Health and Safety, Maisons-Alfort, France; ^2^Santé publique France, Direction des régions, Bretagne, Saint-Maurice, France; ^3^HQSA, EnvA, Maisons-Alfort, France; ^4^UMR INRAE 1014 SECALIM, Oniris, Nantes, France

**Keywords:** COVID-19, SARS-CoV-2, meat processing plant, risk factors, mitigation measures, occupational health

## Abstract

**Introduction:**

Several COVID-19 outbreaks have been reported in meat processing plants in different countries. The aim of this study was to assess the environmental and socio-economic risk factors favouring the transmission of SARS-CoV-2 in meat processing plants and to describe the prevention measures implemented.

**Methods:**

Data from epidemiological investigations of COVID-19 clusters in France, the scientific literature, structured interviews and site visits were collected and summarised to investigate the main risk factors for SARS-CoV-2 infection in meat processing plants, including determinants within and outside the workplace.

**Results:**

An increased risk of infection was identified among workers with unfavourable socio-economic status (temporary/non-permanent workers, migrants, ethnic minorities, etc.), possibly related to community activities (house-sharing, car-sharing, social activities). Working conditions (proximity between workers) and environmental factors (low temperatures and inadequate ventilation) also appear to be important risk factors. These environmental conditions are particularly prevalent in cutting and boning plants, where the majority of reported cases are concentrated. Preventive measures applied included screening for COVID-19 symptoms, testing, wearing masks, increased hygiene and sanitation, physical and temporal distancing, control of ventilation. Certain food safety hygiene measures were compatible with protecting workers from SARS-CoV-2. The hygiene culture of agri-food workers made it easier to implement preventive measures after adaptation.

**Conclusion:**

This study made it possible to identify the environmental and socio-economic factors conducive to the transmission of SARS-CoV-2 in meat processing plants. The knowledge gained from this work was used in simulations to understand the transmission of the virus in the plants.

## 1 Introduction

During the COVID-19 pandemic, many countries imposed national lockdowns to limit the spread of SARS-CoV-2. This measure restricts travel to an absolute minimum. Food processing plants have been identified as critical infrastructure. Workers in the agri-food sector (from production to processing and distribution) were among the critical workers, as they enabled the production chain to function.

In France, where the food industry represents more than 17,000 companies and 400,000 jobs, the first lockdown took place from 17 March to 10 May 2020. Two additional lockdowns with fewer restrictions were implemented from 30 October to 15 December 2020 and from 3 April to 3 May 2021 (1).

Several COVID-19 outbreaks in the food processing industry were reported in different countries, associated with high levels of SARS-CoV-2 transmission (2–4). These COVID-19 clusters in such a vital sector as the agri-food industry have raised concerns about both the health of workers and the economic impact, with the risk of supply chain disruptions or loss of export markets (some countries require SARS-CoV-2 testing of food products or their packaging). By way of illustration, in France, the meat sector represents around 3,300 companies employing around 100,000 people (5). There are 263 slaughterhouses, 70 of which produce 75% of the total national tonnage.

Some agri-food facilities have been particularly affected, such as red meat and poultry processing plants, for which the US Centers for Disease Control and Prevention (CDC) reported 16,233 cases in 239 facilities in April and May 2020 (6). In France, among 1813 clusters reported in the workplace between May and October 2020, 79 clusters occurred in the agri-food industry (7).

Because of the need to maintain and control the cold chain, working conditions in some food processing plants can be difficult, especially in those with low temperatures and high relative humidity. Indeed, on the one hand, the facilities must allow the maintenance of the regulatory temperature in the workshops, i.e., below 12°C. However, such environments have been found to be conducive to virus transmission (8). On the other hand, the operators have to work in these workshops with strict hygiene rules that are applied on a daily basis to ensure the safety of the products.

The aim of this study was to assess the environmental and socio-economic risk factors favouring the transmission of SARS-CoV-2 in meat processing plants and to describe the prevention and mitigation measures implemented.

## 2 Methods

Data from epidemiological investigations of COVID-19 clusters occurrences in France and worldwide were collected and summarized to investigate the main risk factors for SARS-CoV-2 infection in meat processing plants including determinants within and outside the workplace. In addition, occupational and environmental conditions were investigated through structured interviews and observations in food processing plants.

**Figure 1:**
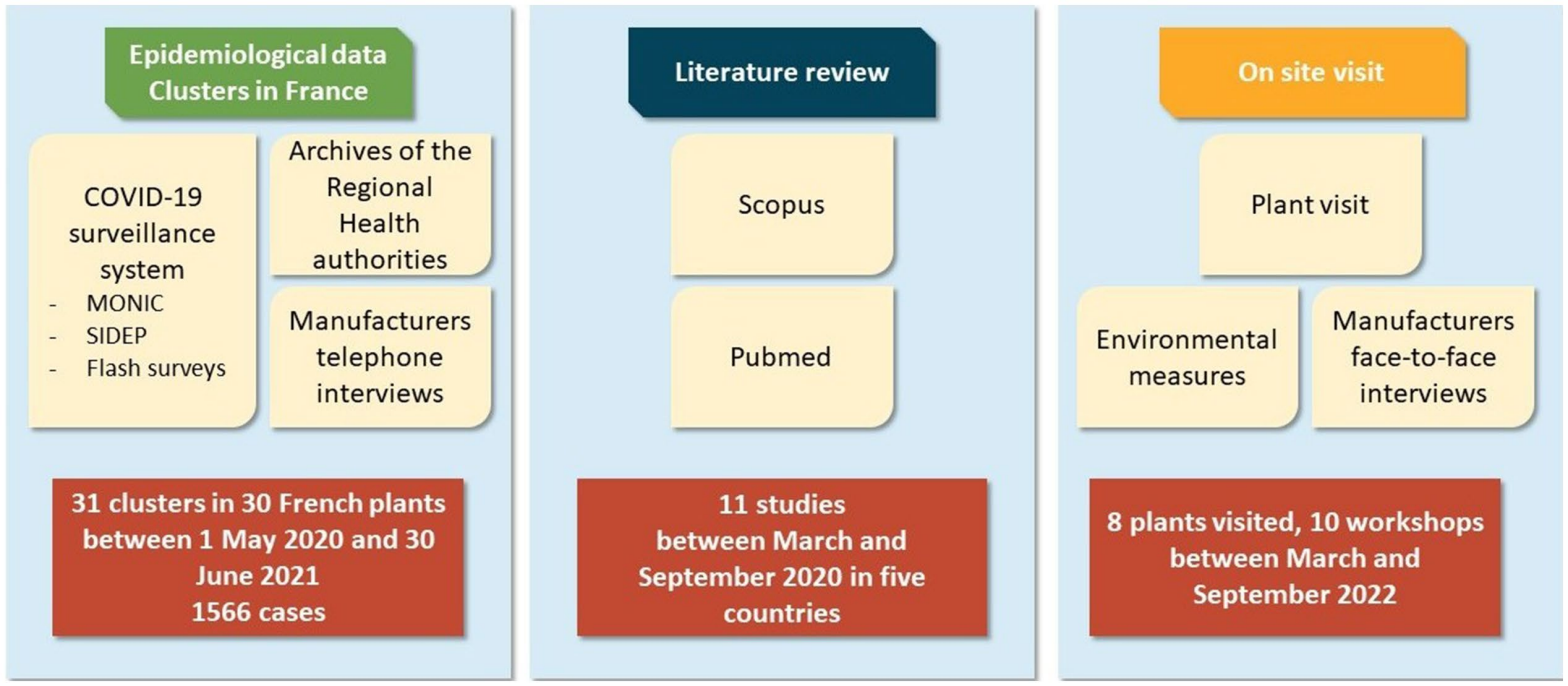
Overview of data sources used in the study.

Three sources of data were used: (1) epidemiological data from clusters occurred in France, (2) a literature review, and (3) on site visits (Figure 1). Data collection and processing are described in more detail below.

### 2.1 Descriptive study of selected clusters occurred in France

#### 2.1.1 COVID-19 surveillance system

In France, biological test results from COVID-19 screening are recorded in a digital platform SIDEP (9). A COVID-19 case was defined as a person with a positive test (RT-PCR or antigen) for SARS-CoV-2 from a nasopharyngeal swab. The SIDEP platform was used to calculate COVID-19 incidence rates from 1 July 2020.

Clusters were registered in a national database called MONIC (MONItoring of Clusters) from May 2020 until the summer of 2021 (7). A cluster was defined as a grouping of at least three cases within 7 days who belonged to the same community or had attended the same gathering.

In addition, the spread of SARS-CoV-2 variants was monitored since February 2021. The viral genome was sequenced from positive samples collected randomly during repeated surveys (flash Surveys).

#### 2.1.2 Cluster selection and study period

Clusters in meat processing plants with 25 cases or more were described if they were reported in mainland France between 1 May 2020 and 30 June 2021. Only establishments with cutting lines were described, regardless of the presence of slaughtering and tertiary processing activities.

#### 2.1.3 Data collection

Cluster identification was carried out using the MONIC database and the archives of the Regional Health Authorities (“Agences regionales de santé,” ARS). The epidemiological context, the plants (type of activities, number of workers), the cluster investigation methods and the socio-economic and demographic characteristics of the workers were retrieved using a standardized data collection tool. Data were collected from the COVID-19 surveillance system, ARS archives and interviews with some affected manufacturers.

#### 2.1.4 Data analysis

The investigation of SARS-CoV-2 transmission chains can be based solely on the contact-tracing strategy (contact isolation and testing) or in combination with screening campaigns (9).

The number of cases was considered reliable for clusters investigated by multiple screening campaigns or where at least half of the plant workers were tested. A cluster with a reliable number of cases was defined as circumscribed if at least ¾ of the cases were clustered in a specific work area (slaughtering, meat cutting, tertiary processing activities, other).

The geographical and temporal distribution of clusters, investigation methods, cluster sizes and plants were described. Attack rates within the plants and within the circumscribed clusters were calculated when case numbers were reliable. It is worth noting that the attack rates were approximated by dividing the number of cases by the total number of workers (rather than tested workers) when test completeness among workers was less than 85%.

Descriptions of the socio-economic characteristics and occupational environment of the cases were made when clusters were circumscribed or when index cases were grouped in a same work area. Such a selection excluded the majority of late-reported clusters which were less relevant for assessing risk factors for SARS-CoV-2 transmission specific to meat processing plants.

Data analyses were performed in R version 4.1.3 (10), using the Chi-square (or Fisher when appropriate) and Wilcoxon tests for comparisons of variables. Measures of association (prevalence ratios) between well-documented variables and SARS-CoV-2 infection were computed with the epitools package (11).

### 2.2 Systematic review of epidemiological studies

The review question concerned the risk factors for acquiring SARS-CoV-2 infections by food workers in meat processing plants. The review question has a typical PECO structure (population, exposure, comparator, and outcome as key elements) and can be broken down, as follows:

- *Population*: cases of COVID-19 in meat processing plants
- *Exposure*: environmental exposures or sociodemographic characteristics
- *Comparator*: Individuals (food workers) free of disease
- *Outcome*: COVID-19 attack/prevalence rates or measures of association between disease and suspected risk factors (odds ratio (OR), risk ratio (RR), prevalence ratio (PR))

Two bibliographic databases (PubMed and Scopus) were queried in August 2021 and updated in April 2022. Searches were conducted using a combination of keywords linked by the logical operator AND: (SARS-CoV-2 OR covid-19 OR coronavirus OR “Corona virus” OR 2019-ncov OR “novel coronavirus”) AND (meat OR poultry OR beef OR pork) AND (slaughterhouse OR processing OR plant OR industry) AND (work* OR occupation*) AND (“Risk factor” OR epidemio* OR cluster OR investigation).

The engines were set to search for these terms in the “Text Word” field for Pubmed and the “Title– Abstract–Keywords” field for Scopus, without date restriction. This search resulted in 54 references (after removing duplicates) which were exported into EndNote software. Each reference record was then screened for relevance for inclusion in the review. Inclusion criteria were (1) epidemiological studies investigating COVID-19 clusters in slaughterhouses and meat processing plants, (2) the presence of quantitative or qualitative data, and (3) full text in English and French languages. Studies that did not meet these criteria were excluded from the systematic review. Examples of excluded studies were: (1) studies on clusters other than slaughterhouses and processing plants (e.g., on-farm), (2) general workplace studies, (3) studies on viruses other than SARS-CoV-2.

After the screening steps, the data from the included studies were extracted using a standardised spreadsheet. The extracted data included the relevant study characteristics such as country, study design, study period, population, case definition, nature of the establishment, and working conditions (including work area, temperature, relative humidity (RH), ventilation). In addition, community exposure factors such as commuting, mode of transport, and housing, as well as prevention and mitigation measures, and outcomes (attack/prevalence rates, association measures with suspected or identified risk factors (OR, RR/PR)) were collected.

### 2.3 On site visits and interviews

Descriptions of the meat processing plants and the various steps can be found, for example, in the sector’s professional documentation (technical guides and guides to good hygiene practice) (12). The Regulations also set maximum temperature limits for products or premises, depending on the production stage (13). However, the scientific literature does not adequately describe and provide a complete overview of the environmental conditions in these plants.

Ten production sites were visited in mainland France between April 2021 to August 2022. The plants visited cover three sectors of the agri-food industry: red meat (beef, and pork for four plants), composite dishes (two plants), and poultry (one plant with slaughtering and cutting activities). These last three plants were included in the visit programme due to their similarities with meat processing plants in several aspects. All of these plants operated below +12°C and respected the three main hygienic principles of design and operation (go-forward movement, zoning, no crossing of circuits). For the meat sector, only plants with cutting lines were visited. Clusters of 25 cases or more were identified in three of the 10 production sites visited.

The purpose of the visit was to learn more about their hygiene management practices and to observe their operations and the effective implementation of preventive or mitigating measures. Interviews were conducted with operators, such as quality managers at eight sites. A semi-structured questionnaire was used to collect information on the general context of work in a food processing plant and on the main changes that occurred during the COVID-19 crisis. The questionnaire included questions (see Supplementary material 1) on the layout of the plant (physical dimensions and environmental parameters of the different areas of the plant), the general operation of the plant (e.g., number of production shifts, number of workers per shift, working hours, movements within the plant, etc.) and the description of the working day (e.g., transition between the changing rooms and the workstation, distances between the workers, the type of masks used, organisation of the breaks).

Additional and specific investigations (questions, measurement campaigns) were carried out to gain a better understanding of the working environment (dimensions, proximity of workers, kinetics of the actual temperature or humidity; see Supplementary material 2).

## 3 Results and discussion

**Figure 2:**
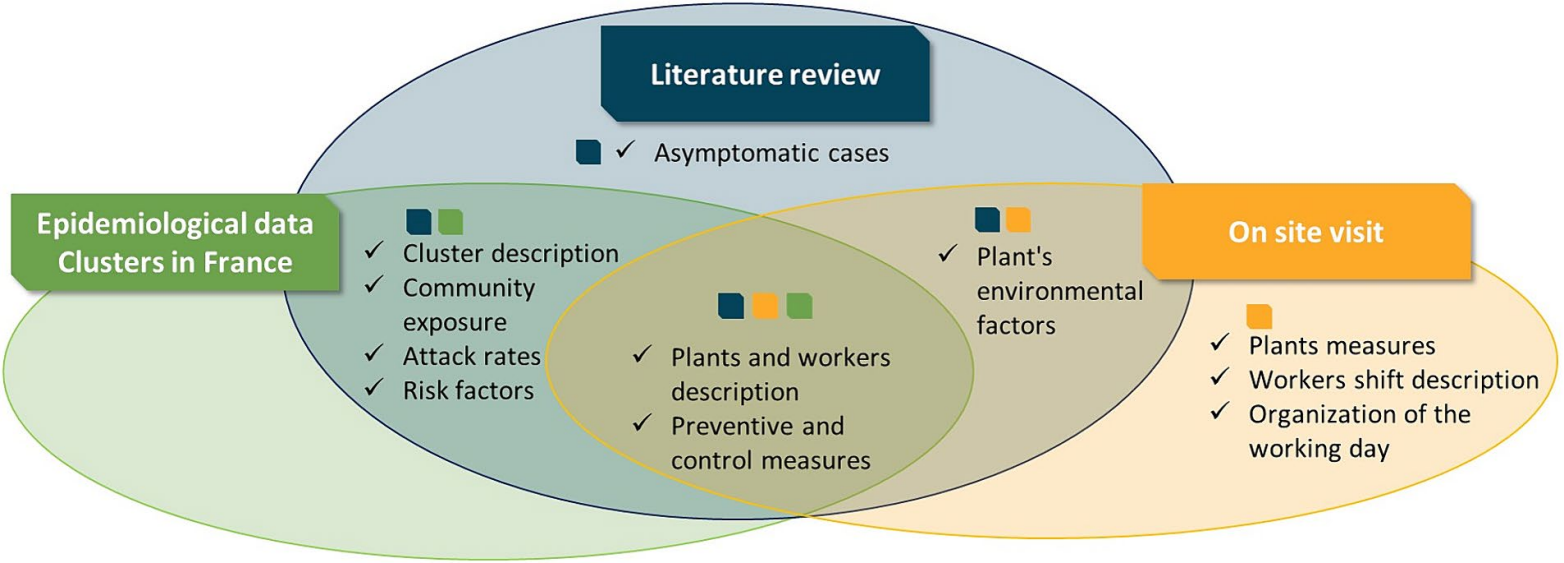
Extracting information from different data sources.

The three data sources complemented each other and provided the necessary information needed to understand the dynamics of clusters in meat processing plants. The contribution of each source is summarised in Figure 2.

### 3.1 Overview of COVID-19 clusters in meat processing in France

#### 3.1.1 Epidemiological context in mainland France

**Figure 3:**
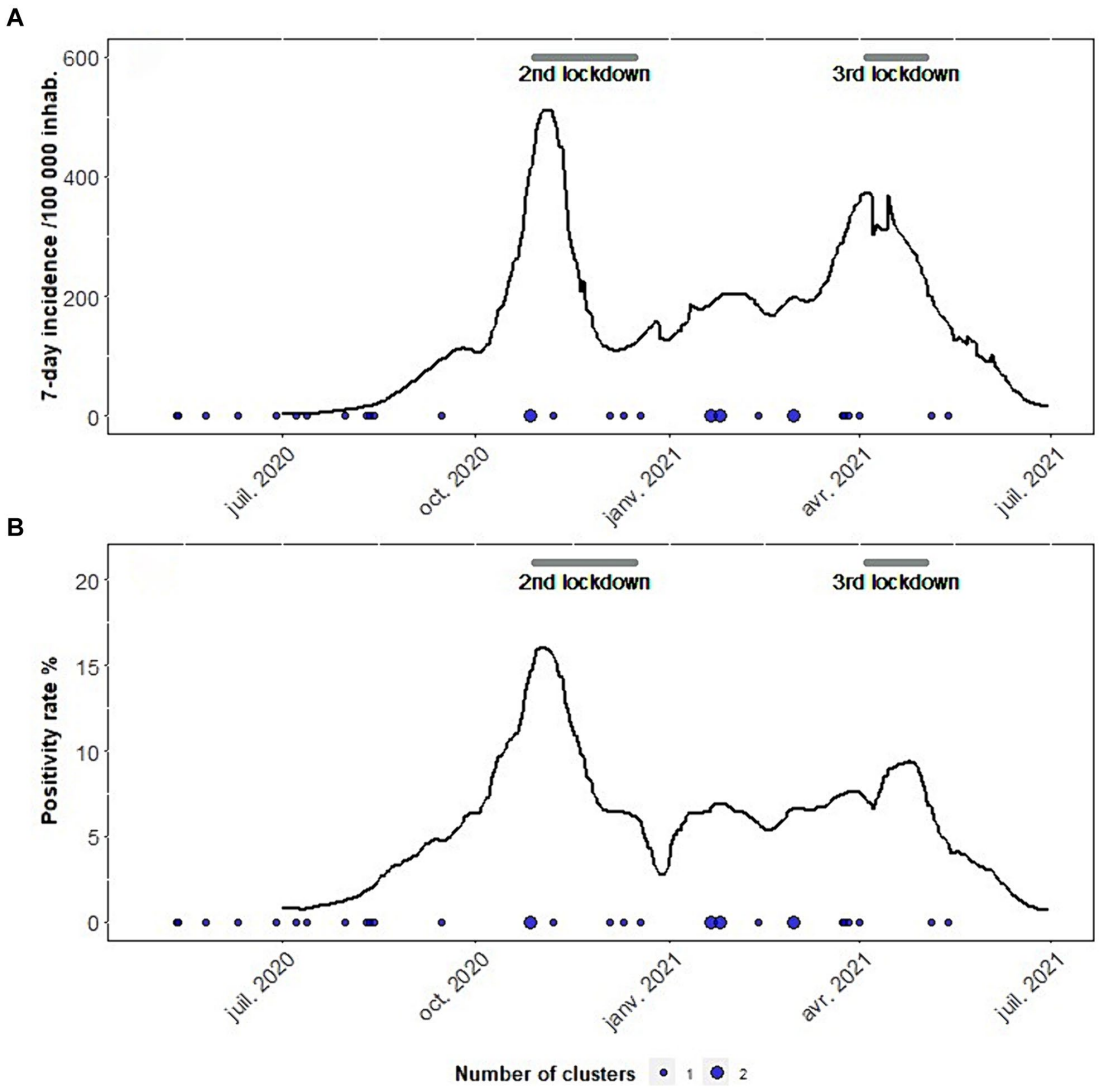
Temporal distribution of the selected clusters (*N* = 31) and COVID-19 epidemiological context (**A** – 7-day Incidence, **B** – Test positivity). Mainland France, from 1 May 2020 to 30 June 2021.

Following the first COVID-19 lockdown, the beginning of the study period was characterised by low incidence rates (Figure 3). The median over the period of 7-day incidence rates was 147/100,000. Incidence peaks in November 2020 (512/100,000) and April 2021 (374/100,000) resulted in the two additional lockdowns.

The last peak occurred after variant Alpha became the majority of interpretable sequences in March 2021. By the end of the study period in June 2021, variant Delta was supplanting Alpha.

#### 3.1.2 Characteristics of the selected clusters

**Figure 4:**
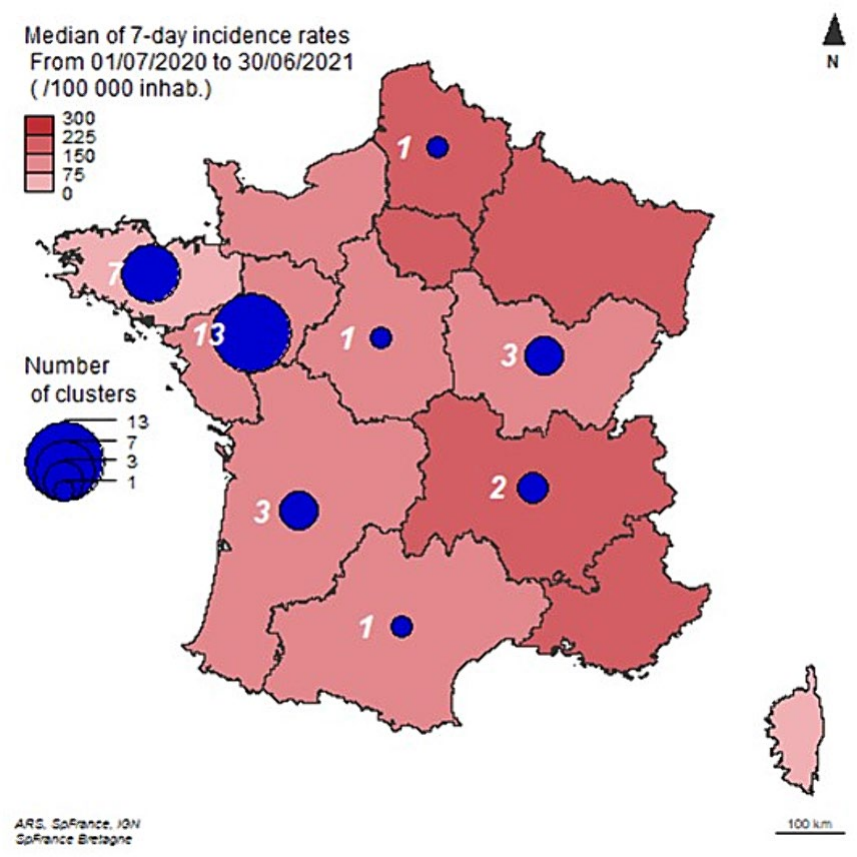
Regional distribution of the selected clusters (*N* = 31) and incidence. Mainland France, from 1 May 2020 to 30 June 2021.

Thirty-one clusters were notified between 12 May 2020 and 13 May 2021: 18 in 2020, 13 in 2021 (Figure 3). They were distributed over eight regions (Figure 4). Twenty of them occurred in northwestern France in the Brittany and Pays-de-la-Loire regions where SARS-CoV-2 circulation was moderate (Figure 4). In these two leading agri-food regions, the median 7-day incidence rates over the period were 74 and 104/100,000, respectively.

Investigation methods were documented for 29 (93.5%) clusters. Of these, four were investigated exclusively through contact-tracing and 25 in association with screening campaigns. One of the latter clusters was documented in more detail and allowed for a cross-sectional description of the workers in terms of socio-demographic and occupational characteristics (9).

Clusters occurred in 30 different plants, 28 of which were involved in slaughtering and meat cutting (hereafter referred to as « slaughterhouses »). The remaining two plants did not have any slaughtering activity. Of these 30 plants, 23 were also involved in tertiary processing activities.

Of the 28 slaughterhouses mentioned above, 16 were poultry plants, and 12 livestock plants. Twenty-three over 28 (82.1%) focused their activities on specific species: chickens (7) and ducks (4) for poultries, cattle (6), pigs (5), and sheep (1) for livestock.

The number of workers, documented for 25 (83.3%) plants ranged from 62 to 1,250. The median number of workers was 362 in poultry slaughterhouses and 539 in livestock ones.

Temporary workers were reported in 25 (96.2%) of the 26 documented slaughterhouses. Subcontracted meat cutters were common in livestock slaughterhouses: six out of eight plants reported subcontractors, accounting for 10.3 to 16.7% of the total number of workers. In contrast, nine out of 10 documented poultry slaughterhouses did not report any subcontracted companies performing meat cutting.

In total, the 31 clusters gathered 1,566 cases. The size of the clusters ranged from 25 to 140 cases: the median number of cases was 44 with no significant difference (*p* = 0.18) between 2020 (49.5) and 2021 (43). Sixteen clusters, gathering 805 cases (51.4%), were circumscribed or had index cases grouped in the same work area. They were all relevant for the additional descriptions.

Attack rates within the plant were assessed for 20 (64.5%) clusters: they ranged from 4.2 to 45.2% with a median rate of 12.2%. Attack rates greater than 20% were observed in four plants with less than 250 workers.

Thus, clusters occurred mainly in slaughterhouses and all animal species were concerned. There was no clear relationship found between the selected clusters and the epidemiological context (incidence rates, spread of variants), either in terms of their distribution (geographical/temporal) or size. However, such relationships could be impaired by differences in adherence to COVID-19 preventive measures among both workers and the surrounding population (1).

#### 3.1.3 Comparison with data reported by other countries

Comparing the French clusters with data reported by other countries is difficult as COVID-19 surveillance system, prevention and mitigation measures (e.g., COVID-19 lockdowns), and the organization of the meat industry were country-specific.

However, it is worth noting that the attack rates in the French plants, ranging from 4.2 to 45.2% (median 12.2%), were consistent with the prevalence rates observed in seven German plants with many infected workers (>10 infected). For the latter, an average prevalence of 10.98% (range, 2.94–35.10%) was reported between late June and early September 2020 (14). In addition, in 122 affected plants of the United States, 10,279 cases of COVID-19 were reported among 112,616 workers corresponding to a prevalence rate of 9.1% (6).

The total number of workers in the French plants are *a priori* reliable. Consequently, the consistency of the attack rates with the literature may suggest that the underestimation of the number of cases (e.g., due to non-exhaustive screening of workers) is low or similar to that observed in other countries.

### 3.2 General characteristics of the studies included in the review

From a total of 54 identified references, 11 studies that met the inclusion criteria were included in the review. One study (15) was first included as a preprint in 2021, and was finally published in 2023.

The studies were conducted between March and September 2020 in five countries: USA (5), Germany (3), France (1), Ireland (1) and Netherlands (1). Table 1 and Supplementary material 3 summarise the main characteristics and the studies. The design of the studies was mainly descriptive (7). Three analytical studies from France (9) and Germany (14, 16) provided a better level of evidence to discuss risk factors for SARS-CoV-2 infection. One of these studies (16) was a retrospective cohort while the last two were cross-sectional (9, 14) and did not allow for a temporal relationship between infection and the factors studied. Finally, one study described COVID-19 cases among workers and quantitatively assessed the impact of mitigation measures (17).

**Table 1:** Characteristics of studies included in the review.

Study ID	Country	Study period	Number of plant	Study design	Epidemiological context	Objectives	Methods
De Rooj_2023	the Netherlands	June 2020	1	Descriptive	Screening campaign (vuluntary basis)	Assessment of potential transmission via air and surfaces in a meat processing plant experiencing clusters	Screening campaign and environmental investigations
Donahue_2020	USA	April–May 2020	1	Descriptive	Preventive screening campaign	Description of the cases (demographic, clinical, household, community and occupational characteristics)	Preventive screening campaign, interview of the symptomatic cases
Dyal_2020	USA	April 2020	115	Surveillance report	Requests from local authorities for COVID-19	Reports of the number of COVID-19 cases across meat and poultry facilities. Facility risk assessments in Meat and Poultry plants—description of the challenges to effective prevention	Description of quantitative and qualitative data (assessment of the cluster and case numbers)
Finci_2022	Germany	April–June 2020	1	Retrospective cohort	Cluster investigation	Description of the cluster, the implemented prevention measures and potential risk factors	Study using data from contact tracing, entry symptom screening, screening campaigns (sero + PCR), the employee roster provided by the employer, the air conditionning construction plan
Gunther_2020	Germany	May–June 2020	1	Descriptive	Large outbreak investigation (>1,400 cases)	Description of the investigation of an outbreak with a focus on the first clustered cases	Contact tracing, screening campaign, viral genome sequencing, employer’s data collection
Herstein _2021	USA	April–July 2020	1	Uncontrolled before and after study (including descriptive data)	Cluster investigation	Description of the cases Assessing the effectiveness of a universal mask policy, and physical barriers	Case description, incidence before and after
Mallet_2021	France	May 2020	1	Cross-sectional	Cluster investigation	Description of the cluster, assessment of socio-demographic and occupational risk factors	Study using data from contact tracing, screening campaigns, employers, case interviews
Pokora_2021	Germany	June–September 2020	1	Cross-sectional	Implementation of a study among selected plants with previous clusters	Identification of risk factors	Description (aggregated data on 22 plants), cross-sectional study (7 plants with “many infected workers”) using data (questionnaires) from the companies and parameters collected on-site.
Rogers_2022	USA	March–June 2020	22/7	Descriptive + cross-sectional KAP (Knowledge, Attitude and Practice) survey + geospatial analysis	Outbreak in a facility	Description of the outbreak assessment of the geographic proximity between community cases and facility cases, description of workers’ knowledge, attitudes and practices	Study using surveillance data at facility (screning campaigns) and in the surrounding community, and results from standardized questionnaires.
Steinberg_2020	USA	March–April 2020	1	Descriptive	Cluster investigation	Description of the outbreak	Contact tracing, screening campaign among symptomatic workers, employer’s data collection, interview of the cases
Walshe_2021	Ireland	March–May 2020	1	Descriptive	Retrospective investigation of a selected cluster	Description of the cluster and risk mitigation measures	Environmental and epidemiological study using data from contact tracing, symptom screening, screening campaigns, site visit / inspection, interview with the Emergency response team, monitoring of air quality.

Most of the studies were conducted in the framework of outbreak investigations or during screening campaigns. Two studies pooled data from multiple facilities (2, 18). Of the included studies, five described the cases in terms of employment status (regular/temporary/subcontracted) or country of origin or primary language (Table 1). Six studies investigated exposures outside the facilities, such as commuting patterns and house sharing. Five studies examined environmental risk factors within the plants (14–16, 19, 20).

### 3.3 Characteristics of workers with SARS-CoV-2 infection

#### 3.3.1 Results from the investigations of French clusters (socio-economic risk factors)

Sixteen clusters were relevant for detailed descriptions in terms of socio-economic characteristics and employers. For seven of these, the data collected (aggregate or line-by-line) allowed comparison between the cases and negative tested workers (Table 2).

**Table 2:** Description of the socio-economic characteristics of cases vs. workers tested negative in a selection of French clusters (May 2020 –June 2021; *N* = 438).

Cluster (*n*)	Median age	Sex-ratio (H/F)	Foreign-born (%)	Subcontracted meat cutters (%)	Temporary workers (%)
Cluster 1^*^ (140)	41 vs. 40 (*p* = 0.31)	2.2 vs. 1.6 (*p* = 0.08)	**52.1 vs. 25.4 (*p* < 10** ^**−3**^ **)**	**45.7 vs. 8.1 (*p* < 10** ^**−3**^ **)**	30.7 vs. 38.3 (*p* = 0.08)
Cluster 2 (52)	41 vs. 43 (*p* = 0.82)	**7.3 vs. 2.0 (*p* = 2.10** ^**−3**^ **)**	**50.0 vs. 15.8 (*p* < 10** ^**−3**^ **)**	NA	**15.4 vs. 3.8 (*p* < 10** ^**−3**^ **)**
Cluster 3 (62)	39 vs. 44 (*p* = 0.07)	3.8 vs. 4.3 (*p* = 0.72)	**45.2 vs. 13.8 (*p* < 10** ^**−3**^ **)**	**71.0 vs. 25.8 (*p* < 10** ^**−3**^ **)**	NA
Cluster 4 (32)	41 vs. 41.5 (*p* = 0.33)	3.8 vs. infinity (*p* = 0.50)	78.6 vs. 85.0 (p = 0.72)	NA	17.9 vs. 0.0 (*p* = 0.07)
Cluster 5 (70)	NA	NA	NA	**44.3 vs. 10.8 (*p* < 10** ^**−3**^ **)**	7.1 vs. 11.1 (*p* = 0.33)
Cluster 6 (54)	NA	NA	NA	31.5 vs. 7.2^**^ **(*p* < 10**^**−3**^**)**	14.8 vs. 27.7^**^ **(*p* = 0.04)**
Cluster 7 (28)	NA	NA	NA	14.3 vs. 2.9 (*p* = 0.17)	NA

*p*-value from the Chi-square (or Fisher if necessary) and Wilcoxon tests for variable comparisons. Statistically significant results are in bold.

* Published investigation (9).

** Cases vs. workers who tested negative or did not test.

#### 3.3.2 Socio-demographic characteristics

Eight investigations (50%) identified carpooling and shared housing among the cases. Six of these reported groups of non-French speaking workers of the same nationality who were heavily affected by the outbreak. Thus, the same group of East-European workers was reported in 5 clusters, including 4 circumscribed clusters. The workers were meat cutters (pork meat on 4 occasions). A second group, from outside of the European Union, accounted for 78.6% (22/28) of the documented cases in an outbreak in a poultry plant.

Three investigations (18.75%) quantified carpooling and shared housing. According to a first investigation, 52.5% (62/118) of the documented cases commuted by carpool or shared accommodation. These risky practices were more frequent among Eastern European workers: 67.3% compared to 39.7% for the other cases (*p* = 5.10^−3^) (9). The other two investigations reported carpooling in 46.8% (29/62) and 50.0% (29/58) of the cases, respectively.

Four investigations (clusters 1 to 4—Table 2), accounting for 316 cases (20.2%), documented cases and workers who tested negative for age, sex and place of birth. Among the cases, the median age was 40.5 years (range, 19–62), the sex ratio (M/F) was 3.3, and 52.8% were foreign born. None of the investigations revealed a significant difference in age between the cases and workers who tested negative. Men were only significantly more present among the cases in cluster 2 (*p* = 2.10^−3^) with a sex-ratio (M/F) of 7.2 compared to 2.0 for the workers who tested negative. In contrast, foreign-born workers were over-represented among the cases (*p* < 10^−3^) in three investigations (clusters 1 to 3), with 45.2–52.1% compared to 13.8–25.4% among the workers who tested negative.

#### 3.3.3 Employers

Six cluster investigations described the cases in terms of subcontracted meat cutters (clusters 1, 3, 5 to 7) or temporary workers (clusters 1, 2, 4 to 6). All investigations, except for cluster 6, compared cases with workers who tested negative. The cluster 6 investigation compared cases to workers who tested negative or did not test.

Subcontractors accounted for 14.3 to 71.0% (cluster 3) of the cases: they were always overrepresented and the excess was significant (*p* < 10^−3^) except for cluster 7 (*p* = 0.17). Only two investigations (clusters 2 and 4) revealed more temporary workers among the cases: the excess was significant for cluster 2 (*p* < 10^−3^).

The association between employers and SARS-CoV-2 infection was assessed only for investigations 1 and 5, which tested more than 85% of the workers. Both clusters occurred in pig slaughterhouses with tertiary processing activities. Cluster 1 had already been described using a multivariable model (9). However, univariate prevalence ratios were calculated in order to compare the association between employer and SARS-CoV-2 infection in clusters 1 and 5. Workers were classified as subcontracted, temporary, and other workers (reference). The prevalence ratio in cluster 1 for the subcontracted meat cutters (5.1 [3.3–7.8]) was close to that assessed in cluster 5 (4.0 [2.7–6.0]). The prevalence ratios for the temporary workers in clusters 1 and 5 were 1.8 [1.1–2.9] and 1.0 [0.4–2.5], respectively.

#### 3.3.4 Comparison with data from the literature

Among the descriptive studies, Steinberg et al. (4) also found that the highest attack rates among non-salaried employees. Donahue et al. (18) also reported that cases were highest among foreign-born or non-native speakers. Two studies (15, 19) reported that many workers (up to 70%) share accommodation and usually commute together to work.

Among the analytical studies, Pokora et al. (14) reported a higher risk of infection for non-regular workers. Finci et al. (16) reported an increased risk of infection for subcontracted employees (adjusted RR = 1.43, 95% CI: 1.06–1.96) which was weaker than that reported by Mallet et al. (9) (adjusted RR = 2.98, 95% CI [1.81–4.99]). In the cohort studied by Finci et al. (16), shared accommodation or transport were common (over 50% of workers). However, these were not identified as potential risk factors for infection among subcontracted employees. Finally, Finci et al. (16) provided an unadjusted risk ratio for a large group of Romanian workers (RR = 3.16, 95% CI: 2.25–4.5) which was close to that reported by Mallet et al. (9) for East-European workers (RR = 4.31, 95%CI: 3.09–6.01).

In summary, the French clusters investigations are consistent with literature data and suggest an increased risk of contamination among subcontracted meat cutters and foreign-born workers possibly related to solidarity practices (carpooling, shared accommodation) and active social lives. These workers with unfavourable socio-economic status may also be more likely to be present at work when sick with COVID-19 (21). They also make up a high proportion of workers in production areas and are more likely to be placed in environments or working conditions that are conducive to virus transmission (see below).

### 3.4 Environmental risk factors

#### 3.4.1 Characteristics of meat processing facilities

**Figure 5:**
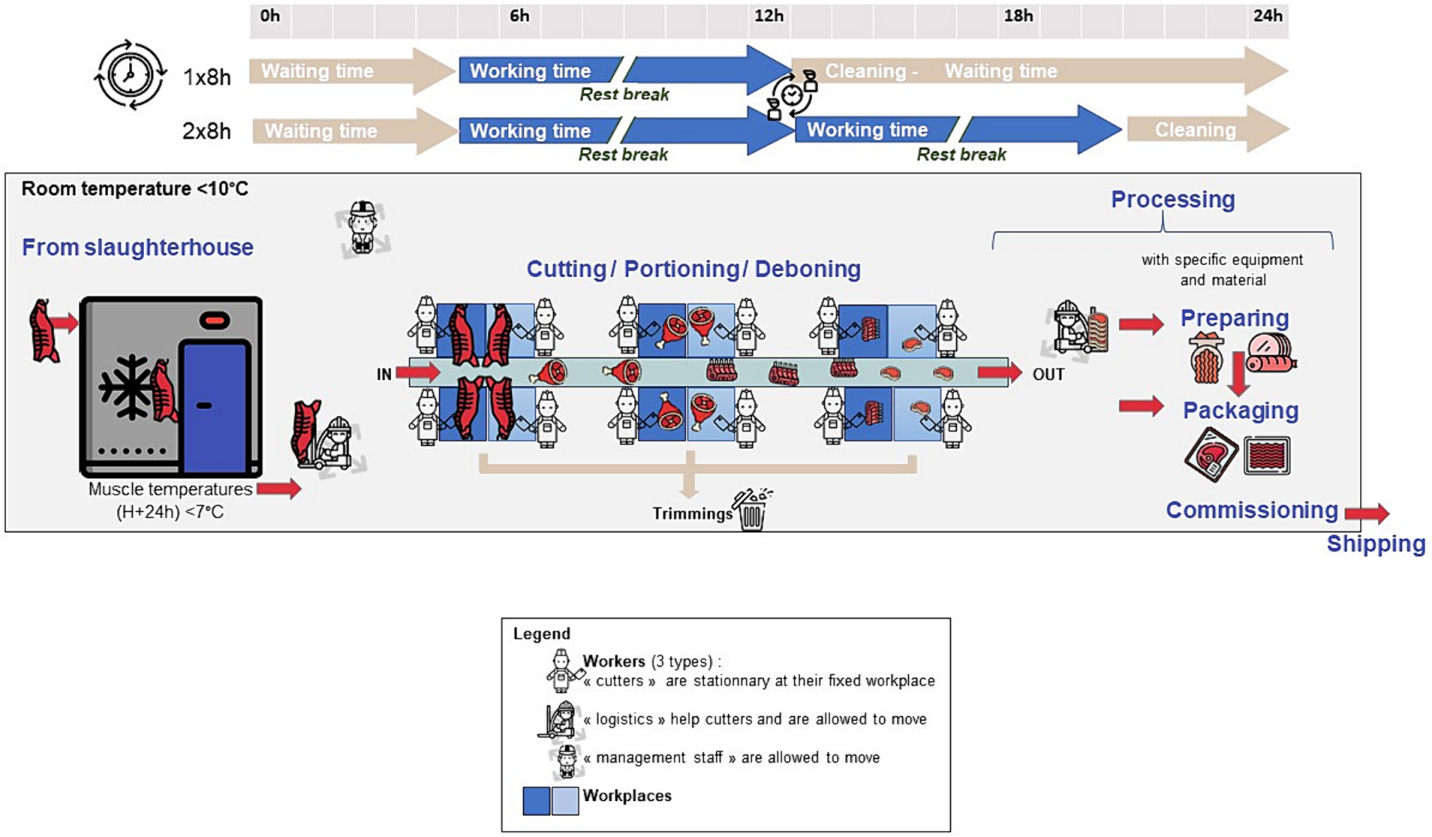
General organisation of a cutting plant (pictograms: flaticon.com).

Visits to the various production sites confirmed the initial hypothesis of a similar mode of operation and organization within and outside the meat products sector. They were designed to ensure a go-forward progressive transition from raw materials (input) to finished or semi-finished products (output) (Figure 5). In general, the organisation and operation aimed to protect the product against all possible contamination from known sources (material, environment, and operators) and then their possible amplification in the product.

Thus, in normal operation (no breakdowns or maintenance), the operators authorised to work in the meat cutting workshops were of three types (Figure 5):

- The “meat cutters,” who worked at the station, did not move except during breaks and generally represented more than 80% of the people present in the workshop during the activity;
- The “logisticians” who helped the meat cutters and moved around the workshop. They represented 15 to 18% of the operators;
- The “management,” who can move around the workshop, was generally represented by 1 to 3 people.

All of these operators were very aware of the application of good hygiene practices. Wearing personal protective equipment such as caps or masks and washing hands and forearms thoroughly were part of the food safety culture. The equipment and materials used in the workshop must contribute to the protection of the product from any contamination by their hygienic design, based on materials approved for contact with food and their high cleaning and disinfection capacity (22). The operator’s role in this matter lies in the selection of hygienic equipment, its maintenance and preventive measures, as well as the design, implementation, and monitoring of a cleaning and disinfection plan (22).

In the vast majority of cases, cutting workshops in France are not cleanrooms (as defined in the standard ISO 14644-1). The control of the air flows is generally partial, the air circulating in a turbulent regime from top to bottom (i.e., from the clean to dirty areas). The circulating air must also be able to reach and maintain a cold ambient temperature of at least +12°C. Two measurement campaigns were carried out in food processing plants to obtain realistic temperature and relative humidity data. The first campaign was carried out in a ready-to-eat meal processing workshop, and the second in a carcass cutting workshop. The observed temperatures were homogeneous and stable over time. The temperature in the cutting room was colder (~7.8°C) than in the ready-to-eat meal processing workshop (~11.7°C). The relative air humidity was also fairly stable over time but showed a wide range of variation, especially in the first measurement campaign (between 45 and 80%). In the carcass cutting room, the relative humidity was observed between 75 and 90%. Temperature and relative humidity conditions were likely to vary from one plant to another. In the sites visited, the hygrometric characteristics were not controlled as shown by the values measured.

Humidity and temperature in production facilities have a major impact on the persistence of viruses such as SARS-CoV-2 (23–25). In meat processing plants, these parameters are also important for the growth of microorganisms found on meat, and thus, for food safety (26).

#### 3.4.2 Environmental risk factors identified during outbreaks

Of the 16 French clusters described in detail, 14 occurred in slaughterhouses and eight were circumscribed. In bovine slaughterhouses, the clusters or the index cases were mainly (6/7) restricted to meat cutting activities. In contrast, five out of seven investigations in poultry plants reported clusters or index cases limited to slaughter. Attack rates were assessed within the perimeter of four circumscribed clusters and ranged from 14 to 34.1%. Several production lines were associated with high attack rates: 31.0 and 44.2% on 2 deboning and ham cutting lines, and at least 52.5% on a poultry hanging and bleeding line.

Attack rates by work area were reported in 10 of the 11 studies reviewed (see Table 3). High attack rates were found in the meat cutting, deboning, and slaughtering areas. Rogers et al. (27) found significant differences in work area between workers with positive and negative SARS-CoV-2 test results. In this study, attack rates were highest among workers in the harvesting area (71%). Finci et al. (16) reported an increased risk of infection in the meat cutting area (adjusted RR: 2.44, 95% CI: 1.45–4.48), which is very consistent with the French investigations including Mallet et al. (9).

**Table 3:** Summary of findings of the studies included in the review.

Study ID	Country	Year /Month	Plant description (meat species)	Work area with higher rate of infected cases	Characteristics of workers (regular/temporary/ foreign)	Risk factors suspected or identified (in bold)
De Rooj_2023	the Netherlands	June 2020	Pig	Deboning and meat cutting areas	100% foreign workers	/
Donahue_2020	USA	April–May 2020	Meat processing facility without further details	Processing (cutting, preparing and packaging meat products)—54%	75% non English-speaking	Close contact with ill person at work
Dyal_2020	USA	April 2020	Beef, bison, lamb, poultry, pork, other	NA	NA	Contact with employee in common areas and outside the facility
Finci_2022	Germany	April–June 2020	Beef, pork	Meat cutting—50.6%, Slaughtering—42.6%, Meat handling in freezer—35.7%, Meat packaging—35.0%	Subcontracters: 70% workers, 43% cases Regular workers: 30.7% workers, 21.7% cases External: 3.3% workers, 18.6% cases	**Subcontracted worker** **Working in the meat cutting area** **Working in slaughtering** **being a veterinary inspector**
Gunther_2020	Germany	May–June 2020	Beef processing plant	Meat cutting area	NA	Environmental conditions distance Close contacts between workers
Herstein _2021	USA	April–July 2020	Beef, pork and poultry primary and secondary processing plant.	Primary processing	NA	/
Mallet_2021	France	May 2020	Pork section	Deboning and meat cutting areas	Temporary and contract workers: 81% cases Foreign born 52% cases	**Subcontracted worker** **Eastern European workers in the deboning and cutting department**
Pokora_2021	Germany	June–September 2020	Meat and poultry processing facilities	Deboning and meat cutting areas, commissioning, slaughter	73% of the study population was temporary or contract workers.	**Minimum distance less than 1.5 meters** **Temperature in working area** **Ventilation system**
Rogers_2022	USA	March–June 2020	Beef	Slaughtering, meat cutting,	NA	Linguistic group Work section
Steinberg_2020	USA	March–April 2020	Probably pork (bacon, sausage)	Slaughtering, meat cutting, processing	The attack rate among nonsalaried employees was 26.8% and among salaried employees was 14.8%.	/
Walshe_2021	Ireland	March–May 2020	Meat processing facility without further details	Slaughtering (Dressing lines) Deboning area	NA	Environmental factors (high occupancy and poor ventilation)

Among the descriptive studies, Günther et al. (19) suggested that low temperature, low air-exchange rates, and airflow and close proximity between workers were factors that could favour aerosol spread of SARS-CoV-2. Their outbreak investigation concluded that environmental conditions promoted viral transmission from a single index case to more than 60% of co-workers within 8 m. Walshe et al. (20) conducted air quality measurements in meat processing areas with the highest COVID-19 attack rates (boning hall and slaughterhouse). Air quality measurements in the boning hall (where 50% of the cases occurred) indicate that carbon dioxide and aerosol particles accumulate over the course of a work shift, indicating poor ventilation.

Among the analytical studies, Pokora et al. (14) reported an increased risk of infection for operators working at a minimum distance of less than 1,5 m (adjusted OR: 3.61; 95% CI 2.83–4.6). The presence of a ventilation system was found to be protective (adjusted OR for the ventilation rate: 0.996, 95% CI 0.993–0.999). Workers in workplaces with higher room temperatures also had a lower risk of testing positive (adjusted OR: 0.90; 95% CI 0.82–0.99).

In conclusion, the French clusters are consistent with the literature data and suggest an increased risk of contamination among workers in the red meat cutting and deboning areas. These areas are characterised by working conditions that favour the spread of SARS-Cov 2, including low temperature, poor ventilation, and lack of social distance between workers. However, the literature review did not confirm that clusters in poultry plants occur mainly in slaughter areas, as suggested by the French investigations.

### 3.5 Preventive and control measures

Several preventive measures have been described in the literature and observed during the cluster investigations and on-site visits. These measures are summarised in Table 4.

**Table 4:** Summary of preventive measures identified in the literature and during the visits and the investigations.

Category	Measures	Details	Limits/challenges
Measures to prevent/limit the entry of infected persons into the establishment			
Early detection of cases	Monitoring temperature and clinical signs	From self-monitoring (temperature, symptoms) to systematic temperature-taking on entering the site. Calling absent workers to rule out the COVID-19 hypothesis.	Temperature measurement at the entrance to the site did not allow asymptomatic workers to be detected: risk of barrier measures being relaxed.
Early detection of cases	Screening campaigns	Unannounced Back from holidays As soon as the first cases appeared	
Isolating cases and contacts	Contact-tracing		Difficult to identify contacts among non-French speakers. Risk of production stoppage.
Distancing	Teleworking	From encouraging teleworking for administrative staff to banning face-to-face meetings	Can not apply to production workers
Distance from the company	Limiting car pooling	Modification of team composition to limit car pooling	
Measures to limit the spread of the virus within the establishment			
Protective equipment	Masks	From fabric mask to FFP2	Availability Renewal Adherence—correct mask wearing
Reinforcement of hygiene measures	Hydroalcoholic solutions	Several points (site entrance, changing rooms, workshops, etc.)	Adherence
	Increased cleaning and disinfection frequency	In the workshops (not specifically or only during breaks) In social premises and communal facilities. (door handles, light switches, etc) Addition of hydro-alcoholic gels at workstations Change of product (use of virucidal products)—verification of the virucidal activity of the products used (request for certificate from suppliers) Tighter controls on cleaning before hiring	
	Operator hygiene	Cleanliness of clothing	
Distancing	Physical barriers	In the workshops (plexiglass) In the social rooms between the tables	Do not prevent aerosol transmission Increased risk of foreign bodies (food safety hazard) Cost
	Physical distance	In changing rooms, toilets and social areas (breaks, catering, etc.): markings on the floor, distance from tables, use of temporary premises, introduction of gauges, changes of use or closure of premises or facilities. In the workshops: bringing unused lines into service or closing a line.	Acceptability—adherence Availability of additional lines Profitability
	Rotation / sequencing	In changing rooms and social areas (breaks, catering, etc.) Within teams, staggered breaks Packed lunches	
Working environment	Air quality	Increase in air exchange rate	Difficulty to maintain in summer
Other measures (communication, monitoring)			
Communication and training		Communication on the symptoms of the disease and preventive measures in different languages Representatives of the different populations could be asked to facilitate the dissemination of prevention messages Reinforcement of training messages	
Monitoring		Monitoring by management of the correct application of barrier measures and distancing rules	Spot monitoring
Vaccination	Implementation of vaccination campaigns		Effectiveness of vaccines in protecting against infection and transmission

These measures were based on various national or international guidelines/ recommendations (28, 29) and have been adapted during the pandemic taking into account the evolution of the scientific knowledge on the virus transmission.

These measures were aimed to (1) prevent the entry of infected persons into the plant (e.g., screening for COVID-19 symptoms, screening campaigns, contact tracing), (2) limit the spread of the virus within the plant (wearing masks, increased hygiene and sanitation, physical and temporal distancing, reinforcement of hygiene measures, increased in-air exchange rate) (20). In addition, communication and training activities were carried out to increase workers’ awareness of the COVID symptoms and the application of preventive measures inside and outside the plant.

These measures have been applied in other occupational sectors and have been found to be effective (29). However, food safety, economic and behavioural factors may limit the application of some measures in meat processing plants (see Table 4). Few studies have quantitatively assessed the effectiveness of these measures in meat processing plants settings. Herstein et al. (17) found that 62% (8/13) of facilities in Nebraska (USA) showed a significant reduction in COVID-19 incidence after implementing both mask use and physical barrier interventions.

## 4 Discussion

The aim of this study was to assess the environmental and socio-economic risk factors that favour the transmission of SARS-CoV-2 in meat processing plants, which are difficult working environments (strenuous work, proximity of workers, low temperature, high humidity) and for which several clusters have been identified. Our study also aimed to identify related preventive measures.

By analysing data collected from epidemiological investigations of clusters in France, the literature, interviews and visits to industrial sites, we were able to highlight the potential risk factors that influence the spread of the virus in these particular environments.

Our results highlighted the importance of several socio-economic factors that need to be taken into account in order to control the transmission of the virus in these occupational settings. The vulnerable populations identified were those most commonly found in the sector (temporary/non-permanent workers, migrants, ethnic minorities, etc.). An increased risk of infection was identified among these groups of workers, possibly related to community activities (house-sharing, car-sharing, social activities) or poor adherence to/understanding of preventive measures (language barrier).

Working conditions (proximity between workers) and environmental factors also appear to be significant risk factors. Among the environmental factors involved in the transmission of SARS-CoV-2 in these occupational settings, temperature and inadequate ventilation were identified as significant risk factors by Pokora et al. (14). Enclosed and poorly ventilated spaces favour the concentration of airborne virus, increasing the chances of exposure and transmission. In addition, cold and damp workplaces favour the persistence of viruses. These environmental conditions are particularly prevalent in cutting and boning plants, where the majority of reported cases are concentrated.

Similar findings on the risk factors for COVID19 outbreaks have recently been reported in meat processing plants in the Republic of Korea (30) and in England (31). Choi et al. (30) also found an increased risk of infection among subcontractors employee (2 times higher), foreign workers (3 to 5 times higher) and workers in carcass cutting areas (5 times higher).

The investigations also explored different types of animal food production, whatever the species (pork, beef, poultry, rabbit). From this analysis, clusters were observed in all types of plants, regardless of the animal species.

A number of preventive measures were identified during the surveys, site visits and in the literature. Integrating all these findings with the existing literature, it is important to emphasise that preventing the transmission of SARS-CoV-2 in workplaces requires a multidimensional approach. Preventive measures must include both (i) environmental interventions, such as cleaning and disinfecting surfaces or controlling ventilation, (ii) interventions related to the applicability of measures, such as the provision of personal protective equipment, (iii) and finally interventions with workers to promote understanding of and compliance with the proposed measures, such as the application of social distancing policies.

The measures identified have also been applied in other occupational settings (29). However, very few studies have quantitatively assessed the effectiveness of these measures.

SARS-CoV-2 control measures can only be effective if they are understood, complied with and applied. Assessing workers’ perceptions of control measures is an important tool for ensuring that these measures are applied effectively. Assessing workers’ knowledge, attitudes and practices would then help to identify the barriers to applying the measures and the levers that enable them to be implemented. For example, following a KAP study of operators, Rogers et al. (27) recommended information measures in different languages, taking into account socio-economic and cultural differences.

The agri-food industry has the dual priority of ensuring both worker safety (through physical activities and possible contamination by biological hazards during production stages) and food safety. The COVID-19 pandemic demonstrated that certain hygiene measures aimed at food safety were compatible with protecting workers from SARS-CoV-2. Compared with other occupational settings, food safety management systems and the hygiene culture of the agri-food workers made it easier to implement control measures such as the use of masks and hand hygiene (32). On the other hand, working conditions in certain areas of the workshop (temperature, overcrowding) made it difficult to implement appropriate preventive measures (e.g., wearing a mask, physical barrier between two workstations). Some of these recommended measures had to be reinvented or adapted to meet the specific needs of workers and food safety.

Our work has some limitations. First, environmental factors have rarely been assessed in studies conducted in France and internationally. Given the climate of global emergency and the number of events or clusters to be covered, this information was not necessarily identified or collected exhaustively. Of the selected studies, only three analytical epidemiological studies were able to identify/confirm risk factors.

In addition, the surveys and interviews used to collect data and information were confronted with the fact that the sectors concerned are also sensitive to other issues in the media. This sometimes made it difficult to obtain descriptive information on the operation of the facilities.

Despite these challenges, all these elements enabled us to gain an overview of the problem in order to identify the environmental and socio-economic factors conducive to the transmission of SARS-CoV-2 in meat processing plants. Our findings are consistent with published reviews of risk factors and preventive measures applicable to the agri-food industry (3, 29, 33).

This work can be integrated into an approach to understanding how workshops operate. To improve our understanding of the transmission of SARS-CoV-2 in meat processing plants, the knowledge gained from this work was used in simulations to understand the transmission of the virus in the plants (34).

By combining these two approaches, it would then be possible to evaluate existing control measures and propose new ones that could be more effective by targeting key transmission routes or identified risk factors.

## 5 Conclusion

In the context of the COVID-19 pandemic, the agri-food industry was faced with three challenges: ensuring the safety of workers, producing healthy food, and securing the food supply. These three elements are interlinked. Ensuring all three was made all the more difficult by the fact that workers could be absent (contact cases), become ill (individually or collectively), or that management measures could lead to the partial or total plant closures.

Fortunately, the food industry has good hygiene practices, that were quickly adapted to prevent the spread of the virus in the production environment. In addition, the hygiene culture of the workforce has made it easier to implement the new control measures.

For the future, it should be emphasised that the investments made by manufacturers in worker safety during the pandemic to protect against SARS-CoV-2 may also improve food safety for other pathogens whose vector may be the worker or the production environment.

## Data Availability

The original contributions presented in the study are included in the article/Supplementary material, further inquiries can be directed to the corresponding author.
