# Supplementary material for: Mitigating COVID-19 in meat processing plants: what have we learned from cluster investigations?
Source: Front Public Health. 2024 Sep 2;12:1432332. doi: 10.3389/fpubh.2024.1432332 (PMC11402690; doi:10.3389/fpubh.2024.1432332)
Supplement: Supplementary file 1 [file Table_1.DOCX]

Supplementary Material

**Mitigating COVID-19 in meat processing plants. What have we learned from cluster investigations?**

**Pauline KOOH***^†^**, Yvonnick GUILLOIS***^†^**, Michel FEDERIGHI, Mathilde PIVETTE, Anne-Laure MAILLARD, Ngoc-Du Martin LUONG, Estelle CHAIX.**

†These authors contributed equally to this work and share first authorship.

***Correspondence:** Pauline KOOH [**pauline.kooh@anses.fr**](mailto:pauline.kooh@anses.fr)

# Questionnaire for interviews and visits to the Transformation Workshops

*This document is a translation of the questionnaire that guided the site visits and interviews. Not all the questions were asked systematically. These elements served as a reference for the interviewer.*

## Questions specific to each room

|  | **Workshop / Cutting room** |
| --- | --- |
|  | **description of space and workers**   - Ratio: number of people per room (volume/dimension) - Production lines: size of conveyor belt and its position in relation to the room - Surface area of cutting station   including a knife sterilizer?   - Are there any other types of posts and where are they located: line manager, administrative staff, logistics staff (bringing in carcasses, moving finished products, etc.)? - Proportion of job types : how many workers (%) are cutters, logistics, etc.)? - Presence of other equipment: vacuum sealer, tray packer, peeler, etc. |
|  | Are there any recommendations for ventilation and air renewal (e.g. 50% fresh air)? |
|  | Is there excess pressure in the workshop? |
|  | What is the target temperature? |
|  | Is the humidity controlled, and if so, what is the set point? |
|  | Hygiene of the premises :   - Do you have areas inside the workshop that are cleaned more frequently (door, cutting tables, floor, door)? When is cleaning/disinfection carried out? Is it done in-house or outsourced? - If there are polypropylene cutting tables, is there a special cleaning procedure? - "Has the crisis changed the way your premises are run/cleaned (specific measures before/after COVID-19)? |
|  | **Cold rooms for finished products air speed?** |
|  | - dimensions and position in relation to the room? - air speed? |
|  | **Changing rooms** |
|  | How many changing rooms?   - Dimensions / Volume / Position in relation to the room? |
|  | Type of ventilation ? [standard Continuous Mandatory Ventilation (CMV) ?] |
|  | Target temperature in the changing rooms? |
|  | Has the COVID-19 crisis affected the way your premises are run/cleaned? |
|  | Direction of movement in changing rooms   - separation of street clothes and work clothes? |
|  | Entrance to the workshop :   - Is the hire time the same for all operators? - Arrival times and changing times in the changing rooms? - Dimensions / Volume / Position in relation to the room? |
|  | **Break room** |
|  | How many break rooms are there? |
|  | Has the COVID-19 crisis affected the way your premises are run/cleaned? |
|  | Is the break room shared with other areas of the plant?   - Is there a company restaurant? |
|  | Dimensions / Volume? |
|  | Type of ventilation (standard CMV?) |
|  | - Target temperature in the break room? - Radiator heating? |
|  | **Sanitary** |
|  | Number of toilets? |
|  | Are the toilets shared with other areas of the plant? |
|  | Dimensions / Volume? |
|  | Type of ventilation (standard CMV?) |
|  | Target temperature in the toilets ? |
|  | How often is cleaning carried out?  - differences before and after the COVID-19 health crisis? |
|  | **Airlock entrance** |
| 1. 1. | Is there a specific airlock for entering and leaving the cutting plant? |

## General operation

|  | What is the daily hourly shift? 2x8h or 3x7h + 3 hours cleaning?   - Working hours (e.g. morning shift: 5am-1pm; afternoon shift 1pm-9pm)? - Are there alternating shifts per week (e.g morning shift every other week)? |
| --- | --- |
|  | Number of workers per shift in the cutting plant (a range can be given, e.g. 10 to 30)? |
|  | What percentage of non-permanent staff? |
|  | - Is there an operator/m² ratio for the workshop? - Has this ratio changed before, during and after COVID-19 health crisis? |
|  | Do the morning and afternoon teams have the same changing room? |
|  | Is there a time of day when there is a high concentration of people in the same place (e.g. changing room)? |
|  | Do operators change shifts regularly? |
|  | What percentage of operators use   - car sharing? - shared accommodation? - community activity outside the workshop? |
|  | What is the employee turnover (indication of the compliances of barrier gestures, etc.)? |
|  | - Are all the operators dedicated to the cutting workshop? - What % of operators are multi-skilled (a new operator will join the team as required). |
|  | Are the breaks staggered? sequential? in what space? |

## Course of the day

|  | **On arrival at the plant** |
| --- | --- |
|  | Are hands washed on arrival at the changing room? |
|  | Do all operators arrive at the same time and share the changing room? |
|  | Duration of dressing time? |
|  | What are the stages between dressing and arrival at the workshop? |
|  | **In the workshop** |
|  | - Do operators have a designated workstation or do they change during their break? - How are workstations allocated? |
|  | - How far apart are operators / workstations? |
|  | - How often should I wash my hands? |
|  | - Is there a ratio between the number of operators and the number of hand-washing units in the workshop? |
|  | What types of mask are used (surgical, FFP2, no mask, etc.)? |
|  | **Break** |
|  | How long is the break? |
|  | - Do operators change outfits for breaks? - Difference before/after COVID-19 health crisis? |
|  | Do all the operators in the workshop take their break at the same time? |
|  | Do teams on break meet teams from other sectors? |
|  | Do operators change clothes at every break? |
|  | - Where does the break take place (e.g. catering room, outside)? - Can there be several break rooms (a % can be given: e.g. 40% of operators in a break room, 60% outside)? |

## Additional information

Additional information, if available

|  | Production yield (average carcass weight, net weight of final cuts, etc.) for different types of meat (pork/beef, etc.)? |
| --- | --- |
|  | Production volume: e.g. number of carcasses per day/per week, ratio between pigs and cattle, ratio between number of carcasses and number of operators, etc. |
|  | Estimated times (waiting times) for pieces of meat in front of operators and in other areas of the workshop (vacuum machine / tray maker, etc.)? |

# . Characteristics and organization of the visited meat processing plants

| **Parameters** | |
| --- | --- |
| **Characteristics of the processing plant** | |
| Dimensions | - The length and width of the different visited plants varied from 17 to 60m. - The height was estimated approximately between 4 and 7m. |
| Temperature | - Ready-to-eat meal processing workshop : 11.7°C (+/-0.7°C) - Cutting room: 7.8°C (+/-0.8°C) |
| Relative humidity | - Cutting room : 79.8 %RH (+/-4.7 %RH) |
| Common objects and equipment | - Conveyors (dimensions of 40 cm to 1m in width and up to 20m in length), - Cutting tables/surfaces (from 40cm to 1m), - Packaging equipment (e.g., vacuum sealing machines, tray maker) - Other specific equipment (e.g., transport cart, skinning and peeling machines, slicing devices, etc.) |
| **Workers and production line** | |
| Number of workers per area | Depending on the dimension and volume of the plants, several possible situations were observed, e.g.:   - in the same plant : 13 workers for a 377 m^3^ workshop and 20 workers for a 557 m^3^ workshop - 25-50 workers in a plant of 17 x 38 m (W x L), - 60 workers in a plant of 30 x 60 m (W x L), - Up to 85 workers in a plant of 21 x 26 m (W x L). |
| Types of workers | - Deboning and carcass processing workers (~ 36%), - Cutting workers (~ 47%), - Transverse workers (e.g., production line managers, maintenance) (~ 9%), - Other logistic workers. |
| Proximity of workers | Depending on the configuration of the plant and the dimensions of the objects (conveyors, cutting tables), the estimated distance between workers can vary from less than 1 m (e.g., in normal functional situations) to more than 2 m (e.g., in social distancing situations). |
| Working organization | Several possible working shifts:   - Morning: 5:00 AM – 1:00 PM (e.g., cutting/processing/logistics) - Day shift: 9:00 AM – 7:00 PM (e.g., administrative/managers) - Afternoon shift: 1:00 PM – 9:00 PM (cutting/processing/logistics) - Night shift: 9:00 PM – 12:00 AM (cleaning/disinfection) |
| Cleaning / disinfection frequency | Daily |
| Working rhythms | Approximate estimation for the different types of workers based on declared production volumes, for example a deboning/carcass processing worker can have a capacity to cut 20% of a pork carcass during one minute into different portions ready to go to the subsequent processing steps. |
